# Vertebral Artery Fenestration

**DOI:** 10.7759/cureus.245

**Published:** 2015-01-30

**Authors:** Alp Ozpinar, Stephen T Magill, Jason M Davies, Michael W. McDermott

**Affiliations:** 1 Neurological Surgery, Oregon Health & Science University; 2 Department of Neurological Surgery, University of California, San Francisco; 3 Department of Neurosurgery, University of California, San Francisco

**Keywords:** vertebral artery, fenestration, vascular, anatomy

## Abstract

Fenestration of the vertebral artery is a rare vascular anomaly that has been observed at autopsy and on angiography. It is most commonly seen in the extracranial segments of the vertebral artery. This congenital anomaly can arise during multiple different stages of embryological development of the vertebral artery. The clinical significance is unclear, but multiple studies have reported association with other vascular anomalies. Awareness of vascular anomalies is crucial to avoid iatrogenic injuries during endovascular diagnostic and therapeutic interventions. Here, we present a case of a patient with an intracranial vertebral artery fenestration that was identified during work-up for a foramen magnum mass.

## Introduction

Vertebral artery fenestration occurs when the vessel lumen is divided into two separate channels that eventually fuse, forming the primary vessel. Autopsy and angiographic studies suggest that the incidence of vertebral artery fenestration is 0.23%-1.95% [1-2]. Although fenestration of the vertebral artery can occur either intra- or extracranially, extracranial fenestration at the upper cervical level is more commonly reported [3-5]. When the vertebral artery is fenestrated, each channel has its own muscularis layer and is lined by a separate endothelium. The term fenestration has been synonymously used with duplication in the literature; however, the two describe different anatomical phenomena [4, 6]. Duplication of the vertebral artery refers to a condition where the vertebral artery has two origins that fuse at different levels of the neck [6]. Whereas fenestration occurs when there is a single origin that splits to form two channels that re-fuse distally. Interestingly, several case studies have suggested that there may be an increased incidence of saccular aneurysms and arteriovenous malformations in patients with a fenestrated vertebral artery [7-9]. Other studies have reported association with epidermoid cysts, persistent trigeminal neuralgia, and agenesis of the corpus callosum [9-10].

Here, we present a case of intracranial vertebral artery fenestration in a patient with a foramen magnum meningioma.

The patient signed an informed consent to use his health information for treatment and research purposes.

## Case presentation

**Figure 1:**
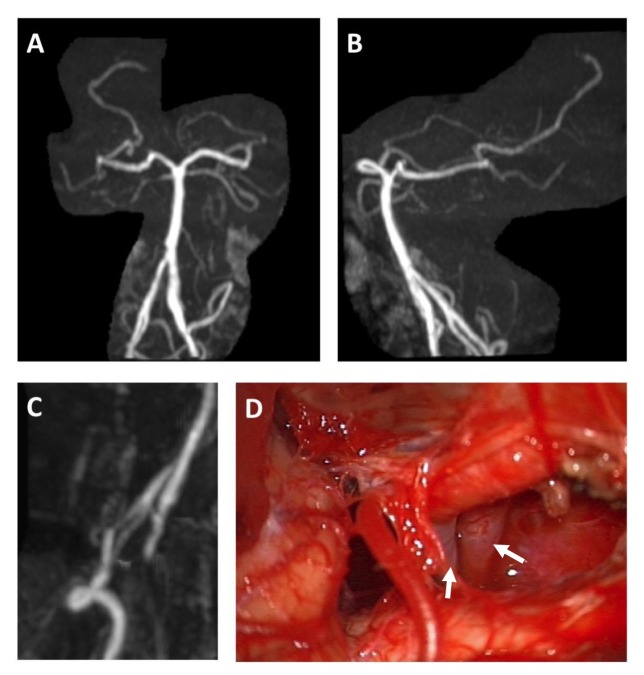
Radiographic and Intraoperative Images of Fenestrated Vertebral Artery MRA time of flight study demonstrating rotated views of the vertebrobasilar circulation. The right vertebral artery demonstrates fenestration in the V4 segment (A-C). Intraoperative photo demonstrating intradural origin and fenestration of the right vertebral artery (D).

A 76-year-old gentleman with no significant medical history presented to our clinic with two years of suboccipital headaches and worsening trapezius wasting. Preoperative MRI revealed a foramen magnum mass intimately associated with the right accessory nerve. The patient did not have evidence of other vascular anomalies. He underwent a far lateral craniotomy and resection of the tumor. The right vertebral artery was visualized intraoperatively with a fenestration extending from the intradural origin at the foramen magnum superiorly towards the vertebrobasilar junction. Postoperative MRI/MRA demonstrated gross total resection of the tumor and confirmed the presence of a vertebral artery with fenestration of the intradural V4 segment extending superiorly toward the vertebrobasilar junction where the two channels eventually re-fused. Figure 1 shows a time-of-flight MRA demonstrating fenestration of the right vertebral artery (Figures 1A-1C) as well as an intraoperative view of the intradural origin of the artery (Figure 1D).

## Discussion

There is significant variability seen in the vertebrobasilar circulation, including duplication of the vertebral artery. Some have postulated that this may be related to the increased amount of collateral circulation [11]. Historically, the terms fenestration and duplication have been inconsistently used throughout the literature, often incorrectly [4, 6]. The term fenestration refers to a vessel with a single origin that divides anywhere along its course into two parallel segments and subsequently rejoins. Duplication refers to a vertebral artery with two origins, a variable course, and fusion [12]. Although uncommon, vertebral artery fenestrations are more prevalent than duplications, and the extracranial portion of the left vertebral artery is the most common reported site for fenestration [3-4, 13].

Embryological development of the vertebral artery is complex. Starting around Day 32 of development, the double dorsal aorta gives rise to seven dorsal cervical intersegmental arterial branches that run with cervical nerves 2-7 [12, 14]. At day 40, the first two intersegmental arteries regress. The third through seventh intersegmental arteries are responsible for the formation of the proximal subclavian and the vertebral artery. The seventh dorsal segmental artery gives rise to the base of the subclavian artery and the proximal vertebral artery. The rest of the segmental arteries involute and give rise to rest of the vertebral artery [12, 15]. Vertebral artery anomalies are thought to be caused by defects in this process of regression and reformation of the intersegmental arteries [16-17]. Multiple theories exist regarding the etiology behind vertebral artery fenestration. Ionete, et al. reported that failure of the regression of the second intersegmental artery is thought to cause extracranial fenestration [6]. Another theory is that the plexiform anastomoses fail to involute, leading to extracranial fenestration [17]. Intracranial fenestration is thought to be caused by persistence of fetal anastomotic vessels, such as Padget’s primitive lateral basilovertebral anastomosis or arteries derived from trigeminal and optic arteries [16-17].

While the clinical significance of vertebral artery fenestration itself remains to be determined, it has been associated with multiple co-morbid vascular malformations. Kubo, et al. demonstrated increased risk of saccular aneurysm formation [9]. Drapkin, et al. reported symptomatic intracranial aneurysm in 20% of patients with vertebral fenestration [3]. Uchino, et al. demonstrated a 7% prevalence of vertebral artery fenestration in 51 cases with known arteriovenous malformation [16]. They also reported that in middle cerebral artery fenestrations there is a higher incidence of aneurysm formation at the location of the proximal bifurcation site [16]. Other reports have shown association of vertebral artery fenestration with epidermoid cyst, persistent trigeminal artery, and agenesis of corpus collosum [9-10]. These studies inherently favor finding an association with other anomalies because patients with these conditions are the ones undergoing vascular imaging. Alternatively, it could be that the rates observed in autopsy studies underestimate the actual incidence due to limited sample size. When a fenestration is observed intra-operatively, it is important that the patient be assessed for these associated vascular anomalies, whether by reviewing prior imaging or obtaining the necessary studies, such as MRA or angiogram.

## Conclusions

Vertebral artery fenestration is rare, with an intradural origin being even less common. The authors herein present a case of an incidentally found intradural right vertebral artery fenestration in a man who presented with a foramen magnum mass. Fenestrations are an important anatomical variant to appreciate in order to prevent any iatrogenic injuries while caring for patients undergoing endovascular and invasive intracranial interventions.
